# “If there are no female nurses to attend to me, I will just go and deliver at home”: a qualitative study in Garissa, Kenya

**DOI:** 10.1186/s12884-019-2477-2

**Published:** 2019-09-10

**Authors:** Coralie N’Gbichi, Abdhalah Kasiira Ziraba, David Wainaina Wambui, Pauline Bakibinga, Isaac Kisiangani, Pauline Njoroge, Rumana Noor, Ngugi Njoroge, Raha Abdi Salah, Elmi Mohamed

**Affiliations:** 1 African Population and Health Research Center (APHRC), P.O. Box 10787, Nairobi, 00100 Kenya; 2 Department of Public Health, Brody School of Medicine, East Carolina University, North Carolina, USA; 3 Sister Maternity Home, P.O. Box 545, Garissa, 70100 Kenya; 4 Preventive Healthcare and Epidemiology Consultancy (PHCEC), P.O. Box 639, Wajir, 70200 Kenya

**Keywords:** Barriers, Sociocultural factors health facility delivery, Skilled birth attendance, Kenya

## Abstract

**Background:**

The North Eastern region in Kenya experiences challenges in the utilization of maternal and newborn health services. In this region, culture and religion play a major role in influencing healthcare seeking behaviour of the community. This study was conducted to (i) understand key inherent barriers to health facility delivery in the Somali community of North Eastern Kenya and (ii) inform interventions on specific needs of this community.

**Methods:**

The study was conducted among community members of Garissa sub-County as part of a baseline assessment before the implementation of an intervention package aimed at creating demand and increasing utilization of maternal and newborn services. Focus group discussions and key informant interviews were conducted with clan leaders, Imams, health managers, member of the county assembly, and service users (women and men) in three locations of Garissa sub-County. Data were analysed through content analysis, by coding recurrent themes and pre-established themes.

**Results:**

Using health facility for delivery was widely acceptable and most respondents acknowledged the advantages and benefits of skilled birth delivery. However, a commonly cited barrier in using health facility delivery was the issue of male nurses and doctors attending to women in labour. According to participants, it is against their culture and thus a key disincentive to using maternity services. Living far from the health facility and lack of a proper and reliable means of transportation was also highlighted as a reason for home delivery. At the health facility level, respondents complained about the poor attitude of health care providers, especially female nurses being disrespectful; and the limited availability of healthcare workers, equipment and supplies. Lack of awareness and information on the importance of skilled birth attendance was also noted.

**Conclusion:**

To increase health facility delivery, interventions need to offer services that take into consideration the sociocultural aspect of the recipients. Culturally acceptable and sensitive services, and awareness on the benefits of skilled birth attendance among the community members are likely to attract more women to use maternity services and thus reduce adverse maternal and newborn health outcomes.

**Electronic supplementary material:**

The online version of this article (10.1186/s12884-019-2477-2) contains supplementary material, which is available to authorized users.

## Background

Globally, maternal and child survivals had significantly improved over the Millennium Development Goals (MDGs) era in 2015, with global trends showing marked improvements in maternal and child health indicators [1]. Despite this progress, maternal and neonatal deaths are still high in Sub-Saharan Africa (SSA) and South East Asia. In SSA, some countries have registered increasing proportions of skilled birth assistance and downward trends in maternal and neonatal deaths [1]. Child survival has also markedly improved as well as child health service indicators such as measles vaccination [1, 2]. However, these gains have not been homogenous with some countries performing much better than others and observed variations within countries [1]. Efforts for better performance under the Sustainable Development Goals (SDGs) era have to target and bring on board vulnerable subpopulations who face unique challenges in accessing and using health services [3].

Maternal mortality in Kenya is still high despite the government commitment to address the issue. Although there may be no reliable data, available estimates indicate that the trends over the last 15 years show no significant decline. The recalculated estimates for Maternal Mortality Ratio (MMR) in 2003, 2008/9 and 2014 were respectively 505, 520 and 362 maternal deaths per 1000 live births [4]. The greatest challenge in reducing maternal and neonatal deaths in Kenya lies in counties where access to maternity services is low. In fact, improvements in access to and utilization of maternity services in these counties are not well sustained [5].

Garissa is part of the larger North Eastern part of Kenya, semi-arid with the majority of its local residents nomadic. The MMR in Garissa is still high with 646 deaths per 100,000 live births [5]. Compared to many other parts of Kenya, North Eastern Kenya is far less endowed with social amenities including health facilities and face high levels of poverty due to limited economic opportunities. Generally, girls are married at a younger age -in line with the Somali culture- and some undergo female genital mutilation [6]. According to the Kenya Demographic Health Survey report of 2014, in North Eastern Kenya, estimates show that about 43% of pregnant women attend antenatal care (ANC) clinics at least four times [4, 7] and only 32.4% of deliveries are attended to by skilled health personnel [4, 8]. The government’s efforts in removing fees in most health facilities have so far not resulted to marked improvement in uptake of skilled birth attendance (SBA) in Garissa County [9].

Existing literature shows that most of the barriers to health facility delivery are rooted in the local cultural norms and practices [10–13]. Women in North Eastern Kenya encounter significant challenges in accessing general health care including reproductive health services as well as reliable information [10]. In most Sub-Saharan African cultures, women are generally subordinate to men, have limited autonomy and depend on their husband/partner for decision-making in most matters including health care seeking [11, 13]. In North Eastern Kenya, while some cultural practices promote both prenatal and maternal health, some are detrimental and discriminatory against the health of the mother [8]. Some women feel that the services offered at the health facilities are not aligned with their religious and cultural norms such as the choice of the birth position, and the gender of the health provider [10].

Recognizing that in North Eastern Kenya, culture and religion influence health services utilization, it is reasonable to assume that part of the solution of the problem lies there [14, 15]. The clan (Reer) and the Somali customary law (Heer) are traditional institutions that have been used in mobilizing communities for emergency relief and political representation [14, 15]. These however have not been well utilized in the health sector. In order to increase skilled delivery in Garissa, it is critical to engage cultural institutions and other social influencers, while understanding and addressing key inherent health system barriers through the provision of culturally sensitive services at healthcare facilities. Understanding this is critical in designing interventions that are context specific and responsive to the local needs and socio-cultural dynamics of the population.

This paper was part of a baseline assessment conducted prior to the implementation of a package of interventions aimed at improving the utilization maternal and newborn services. The aim of this paper is to provide an in-depth understanding of what the barriers to health facility delivery are from various stakeholders. The information will ultimately feed into enhancing the provision of current and future interventions aimed at improving maternal and newborn health.

## Methods

### Study site and participants

The study was conducted in three locations within Garissa sub-County. Iftin and Township were selected as intervention sites while Madinah was the control site. In each of the three locations, there are community units (CUs) overseen and served by Community Health Volunteers (CHVs) who in turn report to the respective designated health facilities. CUs in the interventions groups were purposively selected based on discussions with the sub-county community strategy coordinator for the Garissa sub-county. The CUs of the control group on the other hand were chosen based on discussion with the Garissa county health management team (CHMT). Iftin location is served by Iftin sub-County Hospital, Township is served by Sister Maternity Home (SIMAHO) and Madinah area is served by Madinah Health Centre.

### Study design

As part of the preparatory activities before the intervention implementation, we conducted a social mapping exercise to identify all key resources and social influencers in the communities. We identified clan elders, Imams, health managers, political leaders and others opinion leaders. We used purposive sampling to select participants as the population is not nomadic in that part of the country. Participants were selected based on different criteria. Using the quantitative data, we identified women of reproductive age (WRA) (15–49 years old) who had been pregnant in the last five (5) years and who had or had not used specific MNH services. They were approached by trained research assistants and asked to participate in focus group discussions (FGDs). In addition to WRA identified through the quantitative data, the target population included social influencers (religious, cultural, political) -based on their position in the community and their role in the projects- male champions (married men) and service providers.

### Data collection

We developed and tested interview guides to obtain information principally on: (i) views on women giving birth in health facilities; (ii) barriers in accessing and using MNH services at the community and health facility level, (iii) suggestions on how to increase MNH services demand; and (iv) quality improvement at health facilities (Additional files 1 and 2). All field interviewers were trained on the study rationale, the objectives, the study approach and the data collection procedures. Interviews were conducted in Somali by trained research assistants Somali native. A note taker worked alongside with the interviewer taking notes. In both key informant interviews (KIIs) and FGDs, after asking a question, the research assistant let the participant(s) respond and then probe if necessary, to obtain further information before moving to the next topic.

FGDs and KIIs were used to explore the views of community members regarding MNH services offered in Garissa. We conducted two FGDs with mothers who had given birth in the last 5 years in each location. One for women aged 15–29 years and the other among women aged 30 years and above. Similarly, we conducted two FGDs with married men, one with men aged 15–29 years and the other with older men (30 years and above) in each location. One KII was conducted with the political leadership Members of County Assembly (MCA) of Garissa sub-County in Iftin. We also conducted KIIs among the clan leaders (Imam and clan elders) and health services managers in each of the three study sites.

The study was approved by APHRC’s internal ethical review committee and by the African Medical and Research Foundation (AMREF), an external ethical review committee.

### Data analysis

Audio files were transcribed in English by native Somali speakers. The transcripts were analysed using content analysis, by reading through the transcripts to code important information. This allow for complete analysis of the transcripts and not focusing on pre-established codes or themes. Two members of the research team reviewed and coded the transcripts. After coding the transcripts, the two members of the research team identified patterns from the data coded and made connections to recurrent themes and pre-established themes from the quantitative survey such as views on health facility delivery, reasons for home delivery. Due to the fact that the target population share the same culture and views, data saturation occurred at the analysis stage. Themes were re-appearing in most transcripts, relying the same information.

Data were coded using QSR International’s NVivo 12 software.

## Results

Table 1 represents the type, distribution, and content of the interviews conducted. Six (6) themes were identified to understand the key inherent barriers to utilization of maternal health services: gender of service provider, availability of service provider, autonomy and decision-making regarding health care seeking, awareness of the importance of using maternity service and knowledge of availability of service, attitude of service provider, and distance/cost to health facility*.* To elaborate on the findings, anonymous quotations were used bearing the participant’s position in the community and the type of interview.

**Table 1:** Data collection summary

Data collection type	Participants	Content of interviews	Sites and numbers
Focus Group Discussions (FGDs)	Women 15-29 years old	-Perceptions on the services received -Challenges they face in terms of child birth and post-natal care -Improvement at the health facility level (equipment, services) -Reasons for home delivery -Partner and TBA presence during delivery	Township (1) Iftin (1) Madinah (1)
	Women 30+ years old		Township (1) Iftin (1) Madinah (1)
	Men 15-29 years old	-Views on health facility for delivery -Recommendation to increase health facility delivery -Challenges experienced	Township (1) Iftin (1) Madinah (1)
	Men 30+ years old		Township (1) Iftin (1) Madinah (1)
Key Informant Interviews (KIIs)	Member of County Assembly	-Policy gaps regarding MNCH in Garissa and potential solutions -Views on engaging TBAs -Challenges	Iftin (1)
	Imams	-Views on health facility delivery -Engaging the community about the importance of health facility delivery -Reasons for home delivery -Recommendations to reduce maternal deaths	Township (1) Iftin (1) Madinah (1)
	Clan elders/leaders	-Views on health facility delivery -Recommendations to increase health facility delivery and increase awareness in the community	Township (1) Iftin (1) Madinah (1)
	Health managers	-Services offered at the health facility -Challenges in providing services at the health facility -Equipment and provision of service -Their views on how culture influences health care seeking behaviour -Solutions to improve services offered at health facility	Township (1) Iftin (2) Madinah (2)

### Gender of service provider

Preference of female over male service providers came out strongly as a key barrier in using maternity services which are often provided by male nurses and doctors. This was in spite of the fact that most participants expressed support of women giving birth at health facilities due to the perceived importance of medical support available to them in case of complications or medical emergencies. To some, the preference of female providers was more than a cultural concern. The presence of male nurses during delivery is something they perceived to go against their religious belief. Therefore, Imams and others strongly discouraged women to give birth at health facilities if they were to be assisted by male nurses/doctors.

*“We and other religious leaders are against the use of male doctors during delivery. It is better to get a female doctor. It is not permitted religiously to use male doctors. It is not good for men to breach women’s privacy. In our opinion we suggest female doctors. Every woman will come to the hospital then. This has caused many women to stay and deliver at home … ” (KII clan Imam 4)*

*“ … It is important for them [women] to use the hospitals but if the hospital only has male doctors even if women are present with her, she should deliver outside but if the female doctors are available and equipment and medication are there, she should use the hospital. I would choose she delivers out of hospital if the doctor is male” (KII clan Imam 1)*

When the respondents were asked their point of view concerning male nurses attending to women during delivery, among those who were directly asked the question, beside Imams who were all against male nurses (3/3), several clan elders (2/3), women (8/18) and men (7/19) were outspoken in stating their preference about the gender of the health care provider. It is important to note that a significant number of men and women did not speak on the issue -maybe because they agreed with another respondent’s idea. Many suggested that the health facilities should allocate male nurses to other duties at the hospital rather than attending women in labour.

*“If there are no female nurses to attend to me, I will just go home and deliver at home” (FGD participant [Women group 15–29 years], Iftin)*

*“A woman should be assisted by another female. It is not right for a man to help a mother deliver. When my wife was delivering, I went into the delivery and she was being assisted by a man and to be honest I didn’t like it … ” (KII clan leader 2)*

The desire to have female nurses seems so entrenched that despite knowing the benefits of health facility delivery and the awareness of the possible complications linked to home delivery, people perceived the presence of male nurses at deliveries as something that will continue keeping women away from such facilities even at the risk of suffering obstetric complications.

*“ … Culture and religion will lead women to avoid delivery in the hospitals where males attend to them. Even though she knows the risks of giving birth at home, she’d rather risk them than have a male attend to her in delivery” (FGD participant [ Men group 30+ years], Iftin)*

Among those who posited that breach of religious teaching and culture norm was a major concern, some believed that women are just shy, and do not want to be attended by male nurses because they are not comfortable with men -other than their husband or partner- looking at their private parts.

*“The good thing about giving birth at home is that no one is going to see your private part (male nurses) … ” (FGD participant [Women group 30+ years], SIMAHO)*

*“I went to the hospital and I found that it was a male nurse- I went back home and my husband brought me back and talked to one of the female nurses. So that’s how I delivered in the hospital assisted by a female nurse. As a mother I don’t want to deliver at the hospital assisted by a male nurse, it is better I deliver at home” (FGD participant [Women group 30+ years], SIMAHO)*

### Availability of service providers

It was mentioned by most interviewees including service providers that shortage of staff in the health facilities was a major challenge in women accessing maternal health services. In some facilities, the number of staff available to attend to women varied greatly between day and night and to the extent of not being available at all to attend to the women in labour.

*“Currently we have four nurses. Three during the day and one during the night due to human resources availability” (KII Health manager 3)*

Lived or shared experiences with regard to service quality often inform of the perceptions which permeate communities, and these impressions to a large extent affect service use. The limited hours of service especially in government facilities is often talked about as a quality issue and indeed results show that women preferred to deliver at home than going to a poorly staffed facility at night as one informant stated:

*“During the day they cover well, but for maternity services the delivery occurs at night. If we do not have night staff, then mothers will opt to deliver at home” (KII Health manager 2)*

Still with regard to quality and staffing levels specifically, a critical view that emerged from the key informant interviews is that staff shortage pushed service providers to offer different services at the same time thereby limiting the quality and number of women they could attend to.

*“The only challenge is the shortage of staff because we have one nurse at the ANC and at the same time they conduct deliveries” (KII Health manager 4)*

From these experiences, for some women specifically, knowing the time they are likely to receive services from the health facilities made them selective with regard to the period of the day they go to seek maternal health services despite their vulnerable condition at the time.

*“At night there are no services we get from here, if you go into labour there will be no doctor to assist you. From 6pm there are no doctors, especially the ones for delivery” (FGD participant [Women group 15–29 years], Madinah)*

*“I once came here at 2am and the hospital was open, but I was told that the nurse wasn’t there, I came back at 3am and I was informed that the nurse wasn’t in yet. I came back at 6 in morning, the nurse was around, and I delivered” (FGD participant [Women group 30+ years], SIMAHO)*

### Autonomy and decision-making regarding health care seeking

Quick decision-making is at the center of efforts geared towards preventing adverse maternal and newborn health events as conceptualized in the three delays model. Women’s autonomy and decision-making came up in the discussions painting a picture of men generally being in charge of decision-making in matters related to health care seeking. Health managers perceived it as a cultural barrier in using health facilities. According to them, men have a great influence on the service their wives get at the health facility and especially on the issue of the service provider’s gender. This point of view was supported by only one clan elder stating that males are the one mainly against male nurses during delivery.

*“We have had instances where the men dictate that it [family planning] should not be given” (KII health manager 4)*

*“Most mothers are not supported by their husbands during delivery and when they are there, they don’t allow male nurses and ask for female health workers” (KII health manager 5)*

*“Most of the time it’s a preference of the husband that makes decisions. Mothers might be okay with male providers” (KII health manager 5)*

*“Religion does not prohibit [male nurses] but there are men who are concerned about male doctors who perform deliveries. They are scared in term of religion” (KII clan leader)*

### Awareness of the importance of using maternity service and knowledge of availability of service

It was reassuring to note that almost all study participants seemed to be aware of the importance of health facility deliveries. It is an important first step in efforts geared towards a change in health care seeking behaviour.

*“It’s good if expectant mothers got to the hospital during the time from pregnancy through childbirth. It’s good for them to stop using traditional midwives” (FGD participant [Men group 30+ years], SIMAHO)*

*“It’s a good idea for women to give birth in the hospital because there are things she may not get if she delivers at home. For example, if she delivers here and complications arise, she will be referred to the general hospital by ambulance.” (FGD participant [Men group 30+ years], Iftin)*

*“When a woman becomes pregnant there are so many services she needs from the hospital. She needs to see a doctor who will have her records and follows her up on the progress.” (FGD participant [Men group 30+ years], Iftin)*

Several respondents felt that there were still some community members who did not understand the importance and benefits of health facility deliveries. Some believed that was due to a lack of information, wrong perception of the health facility delivery, while others mentioned that people are just stubborn.

*“Issue of awareness, perception where mothers perceive delivery at the hospital as bureaucracy, is tedious and costly” (MCA Iftin)*

*“Before people didn’t know much, they preferred to give birth at home and the mother believed she would get more care … ” (FGD participant [Women group 30+ years], Iftin)*

*“Lack of knowledge, they do not know the importance of hospital” (FGD participants [Women group 15-29 years], Madinah)*

Most clan leaders blame this lack of knowledge being a barrier to health facility deliveries on the absence of linkage and cooperation between them and the health facility and/or the Ministry of Health. According to them, a partnership will help create awareness in the community and help them understand the importance of health facility deliveries and the availability of services offered to them.

*“The elders should be united with the health workers. There should be proof of female doctors carrying out deliveries. This can change the situation” (KII Clan leader)*

*“The important thing is to first talk to the imams and make them aware. There are different imams in this area for the different mosques. They are the ones who can communicate these issues-they have the microphone and a large congregation to communicate to. They can do it in religious ways” (KII clan leader 2)*

*“This is the work of the ministry of health. They should initiate such a program. They do not invite us for discussion to create awareness by making use of the elders” (KII clan leader 3)*

### Attitude of service providers

The attitudes of service providers offering maternity services in this study were multidimensional. First, it was noted that most female nurses were inactive and lacked enthusiasm towards attending to pregnant women. The respondents reported that clients felt that the midwives were being disturbed when asked to attend to their needs, which otherwise should be their primary responsibility. A man who accompanied his wife to the health facility explained:

*“My wife gave birth through caesarean section and she went through difficulties. We found her fainting (losing conscious) and when you go to the nurses they will tell you ‘we are coming’ and if you call them more than once they will take it bad and think you are pushing them and they become rude” (FGD participant [Men group 30+ years], Iftin)*

Most of the time, this attitude from the nurses affects women’s confidence in utilizing maternity services as it makes them feel like a burden on the health facility while in reality the health facility should ensure that the needs of these women are met. Secondly, while the discomfort around male provider was very apparent, few women mentioned that male nurses showed more empathy to their plight and were more caring and available compared to their female counterparts.

*“Yes, that is the biggest reason-but when it comes to compassion the male nurses are better than the female nurses. The female nurses tell you to walk around when your baby is coming, they don’t show concern” (FGD participant [Women group 30+ years], SIMAHO)*

### Cost/distance

For women living far from the health facility, distance and availability of transport was noted as a great determinant in accessing health services in a timely manner. The availability of ambulances to transport women to the hospitals was said to be limited to some areas leaving some women to opt for other means of transport such as taxi which was expressed as being very expensive, or donkey cart which was regarded as slow and could possibly delay receiving the services. In addition, the poor economic situation of many women deters them from accessing health services. Some women experienced circumstances where there is money available that can be used to pay for transportation to the health facility, however, they are forced to prioritize on what seems important to them, such as food for the family. This is in the context of prevalent poverty and lack of access to other basic needs such as food, shelter and clean water.

*“If there’s a free car that takes mothers to the hospital, it will help women give birth in the hospital. They cannot afford taxi to go to the hospital … A taxi costs Ksh 300 and that is a lot for some people who can’t afford the three meals, so if you cannot afford the transport you will have to give birth at home” (FGD participant [Women group 15–29 years], SIMAHO)*

*“That’s what we are talking about. A mother who has Ksh 300 cannot take a taxi when she needs money for milk and sugar. So, she will just call her neighbour to help her with the delivery” (FGD participant [Women group 15-29 years], SIMAHO)*

Overall, it was found that men and women were not opposed to skilled birth deliveries. Culture was identified as a key barrier to MNH services utilization, which gives room for a behaviour change to improve MNH health outcomes in Garissa. In such setting, creating and sustaining awareness and collaborating with community leaders who have a strong influence in the community is most likely to achieve the goals.

## Discussion

Overall, there is a strong support for women to use health facility for delivery in order to reduce obstetric complications and deaths. However, various factors influence a woman’s decision for the place of delivery including husbands/partners, mothers or the mothers in-law [12]. Our findings are consistent with previous studies analysing barriers in utilizing health facility deliveries concerning woman’s limited autonomy in decision-making and health care seeking [11–13]. In many settings women rely on their husbands regarding health care seeking for themselves and their unborn child [4]. Many women mentioned that the choice of the place of delivery was mainly their husbands/partners’ decision, while husbands on the other hand argue that the wife is the final decision-maker concerning the place of delivery [12]. Moreover, the decision of having a health facility delivery or skilled birth attendance is often made at the onset of labour, unless the woman had previous health issue requiring a pre-established birth preparedness plan [13].

For many, it is unacceptable for a male other than the husband to be present during delivery and this is the key barrier in using health facility for delivery [10, 12, 13]. The main reason being that women do not want to expose their private parts for anyone to see, especially by the opposite gender and or someone younger than them [12]. Unfortunately, in many instances women in labour do not always have the preferred choice of the health provider at a given health facility, and thus to avoid being attended to by a male provider, many prefer a home delivery with a traditional birth attendant (TBA) [13]. Although some believe that the gender of the health care provider is not a major barrier to health facility delivery, few openly opposed that idea in front of their peers.

The choice to deliver at home -with or without a TBA support- ensures for some women that their privacy will not be violated but also more comfort as they will be helped by someone they are familiar with and trust because they share the same culture and tradition [12].

Many women including those using health facility for delivery believe TBAs have more skills and patience than nurses when attending pregnant women. There is a wide range of complaints about the attitude of some health providers being rude, disrespectful and using abusive words toward mothers [11, 13, 16]. Extremes include being physically assaulted and maltreated while being attended to by the nurses [12, 16]. These perceptions and experiences have resulted in a lack of confidence in health care workers and this discourages mothers from seeking care at health facility due to the poor quality of the services they expect to receive [16, 17].

In addition to the poor attitude and doubting skills of health care providers, respondents also complained about the lack of necessary drugs, equipment and other supplies in health facilities as well as a limited number of healthcare workers [11, 13, 17]. In many of such instances patients are asked to purchase the basic supplies needed in order to be attended to [17]. Health care managers and policy-makers acknowledged that staff shortage and the inadequate competency and training of health care workers are critical barriers at the health facility level in providing quality health service to clients [11]. Staff shortage affects the effectiveness and efficiency of health care workers who then have to attend to many mothers at the same time, thus affecting the quality of care provided [10]. Shortage of staff also affects the service hours resulting in some pregnant women not being able to be attended to [16].

Previous studies indicate distance as another key barrier in using health facility for delivery [10, 11, 13]. The lack of a reliable means of transportation forces some people to rely on taxi or a donkey carts, which are very likely to delay them in reaching the health facility in a timely manner. In addition to the poor state of roads, most health facilities cannot provide emergency transportation to the community they serve, and this result in clients spending a large amount of money for private transport [11]. Despite delivery fees being waived by the government or significantly reduced in many health facilities, for many poor households, transport cost is an additional out-of-pocket expenditure which may influence the choice of place of delivery [18]. In fact, previous studies have shown that pregnant women with money set aside are about four times more likely to deliver at health facility with a skilled attendant [19].

Culturally, in many SSA countries, home delivery is seen as a normal and natural process for any strong woman while health facility delivery is suitable for a woman with pre-existing complications [16, 18]. The lack of knowledge and poor communication between the service providers and the community to address such misconceptions has also been a barrier in the utilization of maternity services [10, 16]. Effective strategies to influence social norms that affect MNH services utilization need to be implemented. Evidence from middle income countries have proven that culturally appropriate approaches increases utilization of health services [20, 21]. Improving community’s knowledge on the benefit of health facility delivery as well as equipping health facilities with functional supplies/equipment, emergency transportation, trained, competent and empathic health care workers are key potential solutions to increase health facility delivery and improve maternal and child wellbeing. Beside creating awareness, women empowerment -financial and educational- can be a critical factor in increasing health facility delivery. Indeed, studies have shown that educated and financial independent women are more likely to be involved in the health care seeking decision-making and six times more likely to deliver in health facilities compared to their uneducated and financially dependent peers [19].

### Study limitations

While we took steps to ensure a good representation of the various community strata, we acknowledge the findings in this study may not be applicable to the entire Garissa county population. The study was conducted in a small urban/peri-urban setting, with a predominantly Somali and Muslim community. The urban/semi-urban population in Garissa town may have totally different attitudes, information and practices compared to their rural peers. However, we believe that the rural perceptions and experiences can only be more entrenched as some of the cultural and religious issues tend to have a firmer grip on the lives of rural than urban residents. It is possible that some respondents could have given socially desirable responses and as such misrepresenting the true picture at population level.

This study being a mixed method study, greater impact would have been made by including the results of the quantitative survey to complement the result of the qualitative survey. However, because this study was derived from the baseline results, we believed it would have been better to compared both baseline and end line quantitative survey rather than the qualitative survey.

## Conclusion

The study showed that socio-cultural factors are critical barriers to health facility deliveries in Garissa. Lack of autonomy and decision-making regarding health care seeking by women, service provider gender preference, service providers’ attitude, in addition to transport cost and availability are identified barriers that can be addressed. Strategies that work hand-in-hand with the community stakeholders as well as improving the birthing experiences at health facilities have potential to increase health care seeking and utilization in such settings.

## Additional files

Additional file 1: Interview assessment form. It was used to assess the level of knowledge and understanding of the research assistant collecting the data. (DOCX 21 kb)

Additional file 2: Interview guide. The interviewer followed the guide to obtain the information needed for the study. (DOCX 25 kb)

## Data Availability

The datasets generated and analysed during the study will be made public and available after 2 years on the APHRC Microdata portal: APHRC Microdata portal

## Abbreviations

AMREF: African Medical and Research Foundation
ANC: Antenatal Care
APHRC: African Population and Health Research Center
CHV: Community Health Volunteers
CICF: County Innovation Challenge Fund
KDHS: Kenya Demographic and Health Survey
KSH: Kenyan Shillings
MCA: Member of County Assembly
MDG: Millennium Development Goals
MMR: Maternal Mortality Ratio
MNH: Maternal and Newborn Health
SBA: Skilled Birth Attendant
SDG: Sustainable Development Goals
SSA: Sub-Saharan Africa
TBA: Traditional Birth Attendant
