# Supplementary material for: “If there are no female nurses to attend to me, I will just go and deliver at home”: a qualitative study in Garissa, Kenya
Source: BMC Pregnancy Childbirth. 2019 Sep 10;19:332. doi: 10.1186/s12884-019-2477-2 (PMC6734258; doi:10.1186/s12884-019-2477-2)
Supplement: Supplementary file 1 — Interview assessment form. It was used to assess the level of knowledge and understanding of the research assistant collecting the data. (DOCX 21 kb) [file 12884_2019_2477_MOESM1_ESM.docx]

**INTERVIEWER'S ASSESSMENT FORM**

**AFYA KWA UKOO 3 FIELD INTERVIEWERS**

**NAME OF INTERVIEWEE: ..........................................**

**DATE OF INTERVIEW: ...............................................**

| **MARKS** | |  |
| --- | --- | --- |
| **MAXIMUM** | **ACTUAL** |  |
| **EDUCATION (**Kenya Certificate of Secondary Education (KCSE) certificate, with a minimum grade of C+ or College education (Cert, Dip, Degree) | 10 |  |
| **KNOWLEDGE OF STUDY SITE** (Garissa sub-County-SIMAHO, Iftin & Madina) | 10 |  |
| **PROFESSIONAL STRENGTH & EXPERIENCE:**  Experience in electronic data collection/or certification in computing skills (5)  Experience in managing field work, data quality, data confidentiality (10)  Experience in quantitative data collection methods (Household surveys) (10)  Experience in qualitative data collection (5)  Experience in any other data collection methods (5)  Interviewing skills/handling respondents/slum safety awareness (10)  Experience in community engagement/ mobilization (5) | 50 |  |
| **PERSONALITY** (consider mental alertness, first impression, mannerism and behavior , interpersonal skills, expression of interest in work) | 10 |  |
| **COMMUNICATION** (consider verbal and non-verbal, written: examine original application) coherence, diction and persuasion) (5)  Fluency in English and Kiswahili, knowledge of local language(s) (5) | 10 |  |
| **ADAPTABILITY AND SOCIAL OUTLOOK** (consider maturity of views, flexibility in outlook) | 5 |  |
| **LEADERSHIP** (consider supervisory skills and experience in staff performance evaluations, demonstration of leadership in work place) | 5 |  |
| **TOTAL MARKS** | **100** |  |

Interviewer's general comments on candidate noting particularly those factors which influenced the award of the above marks.

**NOTE: DURING THE INTERVIEW, PLEASE INDICATE:**

1. Area of residence: _____________________________________________________________________
2. Interest in quantitative or qualitative data collection: ___________________________________________
3. Place of interest to work ( for those with an interest in quantitative data collection): _____________­­­­­­______
4. Availability over the period of work: ________________________________________________________
5. How soon the candidate could start: ________________________________________________________

**CONCLUSION**

1. Is the interviewee suitable? Yes/ No
2. Name of the interviewer _________________________________________ Signed ___________________
3. Remarks: ______________________________________________________________________________

_____________________________________________________________________________

_____________________________________________________________________________

_____________________________________________________________________________

_____________________________________________________________________________

______________________________________________________________________________

______________________________________________________________________________
