# Supplementary material for: “If there are no female nurses to attend to me, I will just go and deliver at home”: a qualitative study in Garissa, Kenya
Source: BMC Pregnancy Childbirth. 2019 Sep 10;19:332. doi: 10.1186/s12884-019-2477-2 (PMC6734258; doi:10.1186/s12884-019-2477-2)
Supplement: Supplementary file 2 — Interview guide. The interviewer followed the guide to obtain the information needed for the study. (DOCX 25 kb) [file 12884_2019_2477_MOESM2_ESM.docx]

**Process evaluation tools, Garissa**

1. **FGD’s for mothers**
2. **KII for political leadership**
3. **KII for Health Services Managers**
4. **KII with clan leaders/Imams**
5. **KII with Other Stakeholders such as – UNICEF, and other big actors in the area.**

**AFYA KWA UKOO EVALUATION STUDY, GARISSA**

**Qualitative interview guide for women of reproductive age, political leaders, health service managers, clan leaders/imams, and other key stakeholders.**

These are qualitative interviews (Focus group discussions and in-depth interviews) and below are topics guide with some open-ended questions you can use. But the key is to probe and gain depth of information around the person’s/peoples experiences and expectations about RMNCH.

1. **FGD’s for mothers**

In this area there is a high burden of maternal and neonatal deaths. We are trying to get a better understanding of how women in this community perceive current RMNCH services and their RMNCH needs.

Halkan , wahaa ka jiro culeys weyn oo ah dhimashada hooyada iyo dhalmada. Waxaan isku dayeynaa inaan fahamno sida ay haweenka beesha u arkaan adeegyada RMNCH ee hadda iyo baahidooda RMNCH.

1. What are your views on maternal and child health services in this community?

Waa maxay ra'yigaaga ku saabsan adeegyada caafimaadka hooyada iyo ilmaha ee bulshadan?

- 1. Probe for both positive and negative views

Wereyso: labadaba aragtiyo wanaagsan ama mida xun.

- 1. For the negative views, what can be done differently?

Wixii aragti xun, maxaa duwan oo la samayn karaa?

- 1. For the positive views, how can they be enhanced further?

Wixii aragtida wanaagsan, Sidee loo sii horumarin karaa?

- 1. Which services for MNCH are you aware of that are offered in this community?

Waa maxay adeegyada MNCH aa ka warqabto in la soo bandhigay beesha?

1. In the community, where would you access information for MNCH services in your area?

Bulshada dhexdeeda, halke baad ka heli kartaa macluumaadka adeegyada MNCH ee degaankaaga?

1. What type of services are offered for MNCH?

Mexey noocyadha adeegyada laga bixiyaa MNCH?

1. Do you attend antenatal care services?

Ma tagtaa adeegyada daryeelka dhalmada kahor?

- 1. Probe: where, why and why not

Wereyso: Xaggee, sababta iyo sababta aan u lahayn

1. What are the barriers to seeking MNCH services?

Maxay yihiin caqabadaha helitanka adeegyada MNCH?

- 1. Probe if not mentioned on:

Baar haddii aan lagu soo sheegin

- - - Physical access

Helitaanka Xarumaha,

- - - Cost of accessing services

Kharashka helitaanka adeegyada

- - - Quality of services

Tayada adeegyada,

- Attitudes of the healthcare workers in the health facilities,

Dabeecada shaqaalaha daryeelka caafimaadka ee xarumaha caafimaadka,

- - Operating hours

Saacadaha shaqada,

- - Male involvement,

ka qayb qaadashada nimanka,

- - Community (family, clan & religious leaders),

Bulsho (qoys, qabiil iyo hoggaamiye diineed),

- - Distance, and security.

Masaafada, iyo ammaanka.

- 1. Do you attend antenatal and maternity care?

Ma tagtaa daryeelka dhalmada ka hor iyo kan dhalmada?

- - - Probe: Why and why not

Baaritaan: Sababta iyo sababta laanta

- 1. Do you attend postnatal care?

Maa tagtaa daryeelka dhalmada kadib?

Probe: Why, where and why not

Xaggee, sababta iyo sababta aan u lahayn

1. What do you think are the possible solutions to the barriers you have mentioned?

Maxaad u malaynaysaa inay yihiin xalka suurtagalka ah ee caqabadaha aad soo sheegatay?

- - If these barriers are corrected or minimized, do youthink mothers would seek MNCH services?

Haddii caqabadahaas la saxo ama la yareeyo, miyaad u maleyneysaa in hooyooyinka ay raadsani doonaan adeegyada MNCH?

1. **KII for political leadership**

There is a high burden of maternal and neonatal deaths in this area. We would like to get your views on RMNCH policies.

Halkan waxaa jira culeys weyn oo ah dhimashada hooyada iyo dhalmada. Waxaan jeclaan lahaa inaan helno ra’yigaaga ku saabsan siyaasadaha RMNCH

1. Are there any policy gaps with regards to Reproductive Maternal Neonatal and Child Health (RMNCH) in Garissa County?

Ma jiraan wax faraaq haga sharci dhalmada hooyada iyo Caafimaadka Carruurta (RMNCH) ee Gobalka Gaarisa?

- 1. Probe: Why are there gaps?

waa mexey sababta *faraqahas*?

How can these gaps be addressed?

Sidee loga *hadli* karaa faraqyaha?

1. Is RMNCH a health priority in Garissa?

Miyuu RMNCH u yahay mudnaanta caafimaad ee Gaarisa?

- 1. Why is it a health priority

Maxay utahay muhiimadda caafimaadka?

1. How much funding is allocated for RMNCH?

Meeqo maaliyadeed ayaa loo qoondeeyey RMNCH?

- - - 1. Probe for funding in immunization, safe delivery etc.

Maalgelinta tallaalka, umulaha badbaadhinkodha

- - - 1. Is the funding adequate?

maalgalinta meey ku filantahay?

- - - 1. Is staffing adequate?

Shaqaalaha miyuu ku filan yahay?

- - - 1. Are the facilities equipped with the necessary equipment for RMNCH?

xarumaha meey qalabeysan yihin qalaba laga maarman u ah RMNCH?

- - - 1. Are the drugs and vaccines well stocked?

Daawooyinka iyo tallaalka si fican miya loo helaa?

1. What are the challenges in attracting healthcare staff to this County?

Mexey yihiin caqabadaha soo jiidanaya shaqaalaha daryeelka caafimaadka gobolkaan?

1. Is this something that is also experienced in other counties? And is it being handled in the other counties/regions

Miyuu yahay wax gobolaha kale laaga dareemo? Miyaa lagu maamulaa gobollada / gobollada kale?

1. Are there any strategies in place to attract healthcare personnel to this county?

Miyeey jiraan xeelado meel ay ku soo jiidaan shaqaalaha daryeelka caafimaadka gobolkaan?

1. Is this an issue that is being discussed at national level?

Mawduucan meey tahay arrin heer qaran loo ga wada hadlayo?

1. How is security concerns addressed for MNCH service provision in this area?

Sidee dhibaatooyinka dhinaca ammaanka loo xaliyaa ee bixinta MNCH adeega oo gobolkan?

1. **KII for Health Services Managers**
2. Please comment on how culture influences health seeking behavior in this County?

Si ixtiraam leh ii sharax noocyaha adeegyada RMNCH ee aad bixiso?

- Probe on:
  1. Services provided

Adegyaada la bixiyo

- 1. Equipment for the provision of RMNCH services

Qalabka loogu talagalay bixinta adeegyada RMNCH

- 1. Facility hours

Saacadaha Xarunta

- 1. Workload on employees

shaqalaha shaqadoda

- 1. Employee remuneration etc.

Mushahaarka shaqaalaha

1. What challenges do you encounter in the provision of the RMNCH services you provide?

Waa mexey caqabadaha aad kala kulantay bixintanka adeegyada ee RMNCH aad bihisan?

- 1. What are the staffing challenges

waa maxaa caqabadaha Shaqaalenta kusabsan?

- 1. Are there any challenges in terms of equipment for RMNCH?

Ma jiraan wax caqabad ah oo ku saabsan qalabka loogu talagalay RMNCH?

- 1. Are there any challenges related to the facility hours?

Ma jiraan wax cidhiidhi ah oo la xiriira saacadaha xarunta?

- 1. Are there challenges related to medication and vaccination such as stock out?

Ma jiraan caqabada la xiriira daawooyinka iyo talaalka sida *badeeco*?

- 1. How about the workload on the employees?

Sidee weeye culeyska shaqada ee shaqaalaha?

- 1. Are the remunerations’ for the health workers competitive?

Mushaharka shaqaalaha caafimaadka miyuu yahay ku fican?

- 1. Are there cultural barriers that affect utilization of RMNCH services you provide? Ma jiraan caqabado dhaqameed oo saameeyn uu leh isticmaalka adeegyada RMNCH ee aad bixiso?
  2. How does the health seeking behavior in the community affect MNCH service provision?

Siduu yahay dhaqanka raadinta caafimaadka ee beesha dhexdeeda u saameeyn adeegga MNCH?

- 1. What are your views on the impact of insecurity on MNCH service provision and uptake? For instance do the staff feel safe to go to the community to provide outreach immunization or ANC services?

Mexey yihiin ra'yigaaga ku saabsan saameynta amni-darradis ee ku aaddan bixinta adeegyada MNCH iyo qaadashada? Tusaale ahaan, shaqaaluhu miyuu dareemaan nabadgalyo si ay u tagaan bulshada ay u bixiyaan tallaalada iyo adeegyada ANC?

1. What in your view would be possible solutions to the challenges you have mentioned

Note to interviewer: Make sure to remind the respondent of all the challenges they have mentioned and ask what the possible solutions to those challenges are.

Ra’yigada, maxay noqon kartaa xalka suurtogalka ah ee caqabadaha aad sheegatay?

Note to interviewer: Make sure to remind the respondent of all the challenges they have mentioned and ask what the possible solutions to those challenges are.

1. **KII with clan leaders/Imams**

This area is known to have a high burden of maternal and neonatal deaths. We are trying to gather information about the reproductive, maternal, neonatal, and child health practices in your community. To avoid pregnancy related complications, it is recommended that pregnant women seek care at health facilities.

Halkan waxaa la ogyahay inay leedahay dhimashada badan oo hooyada iyo dhalanteed. Waxaan isku dayeynaa inaan ururinno macluumaad ku saabsan habdhaqanka dhalmada, hooyada, dhalmada, iyo dhaqanka carruurta ee beeshaada. Si looga fogaado dhibaatooyinka la xiriira uurka, waxay soo jeedisay in haweenka uurka leh ay daryeel caafimaad u raadsadaan xarun caafimaad.

1. What are your views about women delivering in a health facility?

Ra’yigada ku sabsabsan dumarka ku umulayan xatunta cafimadka?

1. Probe for both positive and negative views.

Wereyso: ra’yiga fican iyo ra’yiga xun

1. For the negative views, what can be done differently?

Hadii ra’yi xun sheego, maxaa duwan oo la samayn karaa?

1. For the positive views, how can they be enhanced further?

Ra’yiga fican: sidee loo si wanajini karaa?

1. As an imam/clan leader, when you have the opportunity for instance during your sermons, qutba, or during meetings with the community etc., do you recommend that women deliver in a health facility?

Adiga hogaamiya dinta/qabilka ahan, fursad markad heshid tusaale ahan intaa lagu gud jiro wacdiin amise markii bulshada kulman. Ma gu talisay inay dumarka ku umulan xarumaha cafimadka?

1. What is your recommendation to avoiding pregnancy related complications in order to reduce maternal and neonatal deaths in this community?

Waa mexey taladaada si aan looga fogaadho dhibaatooyinka la xiriira uurka si loo yareeyo dhimashada hooyada iyo dhalmada ee bulshadan?

1. **KII with Other Stakeholders such as – UNICEF, and other big actors in the area.**

We understand in this area there are several challenges in access and uptake of RMNCH which include; cultural, structural, policy and financial challenges.

Waxaan fahamsanahay aagahan waxaa jira caqabado dhowr ah oo ku saabsan helitaanka iyo hagaajinta RMNCH oo ay ka mid yihiin; dhaqanka, qaabdhismeedka, siyaasadaha iyo dhaqaalaha

1. In your view, what are the challenges in the access and uptake of RMNCH in this community?

Aragtidaada, mexey yihiin caqabadaha jiran helitaanka iyo kor u qaadista RMNCH ee bulshadan?

- 1. If not mentioned, probe for: Cultural, structural (e.g. girl child education), policy and financial challenges.

Haddii aan laguu sheegin, wereyso: Dhaqanka, qaabdhismeedka (sida waxbarashada gabadha), sharci iyo caqabadaha maaliyad.

- 1. What in your opinion can be done in this community to alleviate the access and uptake challenges?

Ra'yigaaga, maxaa laga qaban karaa beesha si loo yareeyo caqabadha helitaanka iyo qaadashada?
